# Do Demographics and Comorbidities Act as Predictors of Co-diagnosis of Attention-deficit/Hyperactivity Disorder in Autism Spectrum Disorder?

**DOI:** 10.7759/cureus.7798

**Published:** 2020-04-23

**Authors:** Sahar Ashraf, Noha Eskander, Ozge Ceren Amuk, Rikinkumar S Patel

**Affiliations:** 1 Psychiatry, Mayhill Hospital, Denton, USA; 2 Psychiatry, Ain Shams University Hospital, Cairo, EGY; 3 Psychiatry, Koç University School of Medicine, Istanbul, TUR; 4 Psychiatry, Griffin Memorial Hospital, Norman, USA

**Keywords:** autism, attention deficit hyperactivity disorder (adhd), child and adolescent psychiatry, length of stay, comorbidities

## Abstract

Objective

The study aims to determine the demographic predictors of attention-deficit/hyperactivity disorder (ADHD) in hospitalized children with autism spectrum disorder (ASD) and the impact of comorbidities on the length of stay (LOS).

Methods

A retrospective study was performed using a nationwide inpatient sample from US hospitals. All patients were ≤18 years in age with a primary diagnosis of ASD (n = 3,095) and grouped by co-diagnosis of ADHD based on the International Classification of Diseases, 9th Revision, Clinical Modification (ICD-9-CM) diagnosis codes. Logistic regression was used to calculate the odds ratio (OR) and linear regression for estimated LOS.

Results

Male patients had a higher odds of comorbid ADHD (OR: 2.2). Age and race were not significant predictors of ADHD though the condition was found to be prevalent in adolescents and Caucasians. These children were mainly from the South (30.8%) and the Midwest (29.9%) regions of the US. Psychosis was seen in 37.3% of patients with ADHD and was more likely to be comorbid psychosis (OR: 1.8). Depression and ADHD increased the LOS in hospitals for ASD by 2.1 days and 0.9 days, respectively.

Conclusion

Our study led us to determine the demographic predictors of comorbid ADHD in patients with autism, and we believe that our findings can help to better serve these patients and their families. Comorbid ADHD and depression can prolong the length of hospitalization and they necessitate the need for acute inpatient care in such patients.

## Introduction

Autism spectrum disorder (ASD) is a lifelong, pediatric neurodevelopmental disorder characterized by marked impairments in socialization, communication, and the presence of restricted, repetitive behaviors or interests [1]. It is estimated to affect 1% of the population and is more common in males. Many of them are afflicted by coexisting psychiatric conditions, further compromising the function and quality of life [2].

In children diagnosed with ASD, 59% also suffer from attention-deficit/hyperactivity disorder (ADHD). Even though ASD and ADHD are separate conditions, there is an overlap in their disease presentations [3]. Similar abnormalities in the gray matter volume of ASD and ADHD patients have been observed. This could be one of the possible neurobiological basis for the overlapping symptoms of inattention, poor executive function, hyperactivity, and issues with social interaction [3]. Due to the presence of ADHD symptoms in both conditions, a diagnosis of autism is sometimes delayed or misidentified [4].

This study aims to determine the demographic predictors and the risk of comorbidities of ADHD in patients with ASD in the inpatient population. The demographic matrix plays an important role in behavioral conditions and our results may help the physicians to identify ADHD in autistic children. Further research is required to measure the impact of demographic characteristics and comorbidities on the duration of hospitalization for ASD.

## Materials and methods

Data source

A retrospective analysis was performed using the Healthcare Cost and Utilization Project's (HCUP) Nationwide Inpatient Sample (NIS) data from January 2012 to January 2014 [5]. The Agency for Healthcare Research and Quality (AHRQ) sponsors the HCUP databases, and they can identify hospital utilization patterns and cost in the US. The NIS database is the largest inpatient database available in the US and represents numerous non-federal community hospitals [5].

Sample selection

Based on the International Classification of Diseases, 9th Revision, Clinical Modification (ICD-9-CM) diagnosis codes, we identified the patients (≤18 years) hospitalized with a primary diagnosis of ASD (ICD-9-CM code: 299.00). The primary diagnoses in the NIS refer to the condition for which the patient is mainly managed during the hospitalization stay. The sample population was further divided into two groups (ADHD and non-ADHD) based on ADHD as a secondary diagnosis (ICD-9-CM codes: 314.00 or 314.01).

Variables of interest

In order to measure the differences in demographic characteristics, we included age (<11 and 12-18 years), gender (male and female), race (Caucasian, African-American, Hispanic, Asian, and others), median household income (based on quartile classification) and geographic region (based on the American Heart Association (AHA) annual survey of hospitals) [6]. We evaluated the length of stay (LOS) by calculating the number of nights the patient remained in the hospital for the management of the primary diagnosis [6]. The severity of illness as mentioned in the NIS measured the loss of function [6].

Comorbidities are defined as coexisting conditions in hospitalized patients with ASD and are not directly related to the primary diagnosis, and maybe present prior to the inpatient admission. AHRQ comorbidity software was utilized to generate binary variables [6]. Using ICD-9-CM codes, this variable identified four comorbidities in the records of inpatient admissions: depression (300.4, 301.12, 309.00, 309.1, 311); drug abuse, alcohol abuse (292.0, 292.82-292.89, 292.9, 304.00-304.93 305.20-305.93, 648.30-648.34), and psychosis (295.00-298.9, 299.10, 299.11).

Statistical analysis

An exploratory data analysis was performed using cross-tabulation and descriptive statistics over the NIS to summarize the results. Pearson’s chi-square test and independent-sample T-test were utilized for categorical data and continuous data, respectively. Logistic regression was used to calculate the odds ratio (OR) of the demographic variables and comorbidities in the ADHD group, by keeping the non-ADHD group as the reference category. A linear regression model was used to study the impact of various demographic variables and comorbidities on the LOS. We used the discharge weight variable which is given in the NIS database to obtain the nationally-representative inpatient data [6]. All significance tests were two-sided and a p-value of <0.05 was considered to be statistically significant. Statistical analysis was conducted using SPSS Statistics version 23 (IBM Corp., Armonk, NY).

Ethical approval

To protect the privacy of the patients, physicians and hospitals were de-identified [5]. Our data did not contain any patient identification. Our study did not require approval from the institutional review board as it only includes publicly available NIS data.

## Results

Demographic predictors of comorbid ADHD

We analyzed 3,095 hospital admissions for primary diagnoses of ASD and 32.5% of them had a co-diagnosis of ADHD (n = 1,005). About three-fifths of the patients with ASD were adolescents (12-18 years), yet age was not a significant predictor for a co-diagnosis of ADHD. About 90% of the children in the ADHD group were males and they had two-fold higher odds of ADHD co-diagnosis (95% CI: 1.741-2.749, p: <0.001). The majority of the ASD patients were Caucasians, and gender was not a significant predictor for ADHD in these inpatients. Children from middle-income families (51st-75th percentile) had higher odds for comorbid ADHD and those from high-income families (>75th percentile) were less likely to have a co-diagnosis of ADHD. About 60% of the patients in the ADHD group were from the Midwest and the South, and they were more likely to have a co-diagnosis of ADHD compared to the children with ASD from the Northeast. The differences in demographic characteristics in ADHD versus non-ADHD groups are shown below (Table 1).

**Table 1:** Demographic differences in patients with ASD by co-diagnosis of ADHD P-values of ≤0.05 at 95% confidence interval considered significant. Odds ratio generated by binomial logistic regression model with the non-ADHD group as the reference category ASD: autism spectrum disorder; ADHD: attention-deficit/hyperactivity disorder; OR: odds ratio; CI: confidence interval; SD: standard deviation

Variables	ADHD (-)		ADHD (+)		Regression model		
	N	%	N	%	OR	95% CI	P-value
Age at admission, years							
Mean age (±SD)	11.5 (±4.865)		11.9 (±3.668)		-	-	-
<11	885	42.3	415	41.3	Reference		
12–18	1,205	57.7	590	58.7	1.044	0.896–1.216	0.579
Gender							
Male	1,665	79.7	900	89.6	2.188	1.741–2.749	<0.001
Female	425	20.3	105	10.4	Reference		
Race							
Caucasian	1,300	62.2	670	66.7	Reference		
African-American	305	14.6	165	16.4	1.050	0.850–1.296	0.653
Hispanic	270	12.9	115	11.4	0.826	0.652–1.048	0.115
Asian	65	3.1	25	2.5	0.746	0.466–1.195	0.223
Other	150	7.2	30	3.0	0.388	0.259–0.581	<0.001
Median household income							
0–25^th^ percentile	495	23.7	235	23.4	Reference		
26^th^–50^th^ percentile	490	23.4	275	27.4	1.182	0.954–1.465	0.126
51^st^–75^th^ percentile	450	21.5	280	27.9	1.311	1.057–1.626	0.014
76^th^–100^th^ percentile	655	31.3	215	21.4	0.691	0.556–0.860	0.001
Region							
Northeast	690	33.0	265	26.4	Reference		
Midwest	480	23.0	300	29.9	1.627	1.329–1.99	<0.001
South	535	25.6	310	30.8	1.509	1.236–1.841	<0.001
West	385	18.4	130	12.9	0.879	0.689–1.122	0.301

Comorbidities in ASD patients with ADHD

The difference in the severity of illness between ADHD and non-ADHD groups was not statistically significant (p: 0.965), with the majority of the inpatients having moderate loss of function. Children in the ADHD group had a higher mean number of chronic conditions (3.9 ±1.508) compared to the non-ADHD group (2.9 ±1.451). Psychosis was the most common comorbidity seen (29.9%) in the sample inpatient population (37.3% ADHD and 24.9% non-ADHD). Children with comorbid ADHD had a two-fold higher odds of comorbid psychosis compared to patients without ADHD (95% CI: 1.485-2.090). The difference in comorbid drug abuse and depression was not significant between both groups (Table 2).

**Table 2:** Comorbidities in patients with ASD by co-diagnosis of ADHD P-values of ≤0.05 at 95% confidence interval considered significant. Odds ratio generated by logistic regression model with patients without ADHD as the reference category ASD: autism spectrum disorder; ADHD: attention-deficit/hyperactivity disorder; OR: odds ratio; CI: confidence interval

Comorbidities	ADHD (-)		ADHD (+)		Regression model		
	N	%	N	%	OR	95% CI	P-value
No comorbidities	-	-	-	-	Reference		
Alcohol abuse	0	0	0	0	-		
Depression	100	4.8	55	5.5	1.063	0.995–1.030	0.150
Drug abuse	15	0.7	5	0.5	0.637	0.228–1.782	0.390
Psychosis	520	24.9	375	37.3	1.762	1.485–2.090	<0.001

Predictors of LOS

Children in the ADHD group stayed longer in the hospital (9.2 days ±9.319) compared to the non-ADHD group (7.9 days ±11.971). As per the linear regression model adjusted for demographic variables and comorbidities, ADHD increased the LOS by one day and depression increased it by two days. On the contrary, comorbid drug abuse decreased the LOS by six days. Adolescents had longer LOS, while Caucasians and females had shorter LOS comparatively (Table 3).

**Table 3:** Predictors of LOS in patients with ASD P-values of ≤0.05 at 95% confidence interval considered significant. Estimated LOS generated by linear regression model in patients with ASD ASD: autism spectrum disorder; ADHD: attention-deficit/hyperactivity disorder; LOS: length of stay; CI: confidence interval

Variables	Estimated LOS (days)	95% CI	P-value
Comorbid ADHD	0.939	0.095 to 1.783	0.029
Comorbid depression	2.076	0.266 to 3.885	0.025
Comorbid drug abuse	–5.838	–10.687 to –0.988	0.018
Comorbid psychosis	–0.023	–0.922 to 0.875	0.959
Age of 12–18 years	3.419	2.608 to 4.229	<0.001
Female	–0.344	–1.380 to 0.692	0.515
Caucasian	–2.474	–3.436 to –1.511	<0.001
>50^th ^percentile income	1.135	0.326 to 1.945	0.006

## Discussion

We analyzed the largest inpatient data in the US hospitals and found that boys from middle-income families and those from the Midwest and the South are more likely to have a co-diagnosis of ADHD with ASD. These patients have higher odds of psychosis too. The inpatient LOS for the management of ASD was significantly affected by various demographic characteristics and comorbidities.

The most common age at inpatient admission for patients with ASD and comorbid ADHD was 12-18 years. These disorders were observed at a disproportionately greater rate in boys. The male-to-female ratio is around 4:1 [7]. Males with ADHD tend to display more disruptive behaviors, precipitating more referrals for psychiatric care [7]. In our study, males were more likely to have a co-diagnosis of ADHD, and the male-to-female ratio was the same as seen in a study by Stergiakouli et al. [7]. Caucasian children were diagnosed more with comorbid ADHD as compared to non-Caucasians. This may be because children belonging to minority groups could have limited access to mental health services. There may be differences in parental identification of child mental health problems, need for professional care, treatment benefits, or insurance status, which could contribute to disparities in the use of mental health services [8]. About three-fifths of the total patients with ADHD were from the South and the Midwest and the fewest numbers were from the West (12.9%).

Children diagnosed with both ASD and ADHD had greater treatment needs, more co-occurring conditions, and were more likely to have a combined hyperactive/impulsive and inattentive ADHD subtype [9]. This may be one of the reasons for patients with ADHD in our study to have a longer mean hospitalization stay. Nylander et al. found that patients with ASD were more likely to be diagnosed with psychotic disorders, especially schizophrenia [10]. In our study, comorbid psychiatric disorders existed in both groups, and psychosis was the most common comorbidity. Patients with comorbid ADHD had a two-fold higher likelihood of comorbid psychosis compared to patients without ADHD. Affective disorders, such as depression, are more probable in patients with ADHD [10]. In our study, the difference in comorbid depression was not statistically significant between ADHD and non-ADHD groups. Also, a study by Patel et al. found that cannabis users with ADHD have longer hospitalization stay and incurred more cost, but such impact was not seen in autistic children with comorbid ADHD in our study [11].

As per another recent study, patients with autism had significantly higher LOS as compared to those without autism (6.5 vs. 4.2 days) [12]. And the 2009 Kids' Inpatient Database concluded that hospitalized children with ASD and chronic conditions have longer LOS compared to those without chronic conditions [13]. In our study, we found that depression and ADHD comorbidities, age (12-18 years), and a median household income above the 50th percentile were the significant predictors of longer mean LOS by one to three days.

Our study has a few limitations. The NIS is an administrative database and lacks the patient-level data to make accurate clinical associations. The selection of the patients from the dataset was based on the ICD-9-CM diagnosis codes only and this prevents rater and reporting bias [6]. Despite the difficulty in the diagnosis of both conditions, we were able to form subgroups based on comorbid ADHD based on secondary diagnosis in the dataset. These are the discharge diagnoses in patient records and validated after appropriate inpatient management. Further, such retrospective studies are always subject to selection bias, which might be accentuated by the moderate sensitivity of diagnostic codes for ASD and ADHD. However, the biggest strength of our study lies in the national representation of the dataset, with a uniform collection of data, through ICD-9-CM codes. Also, NIS is subject to minimal reporting bias, and all information is coded independently of the individual practitioner, making it a more reliable source. To our knowledge, this is the first study to report the impact of comorbidities and demographics on the LOS during hospitalization for ASD.

## Conclusions

Identification of ASD and ADHD is becoming increasingly important given their growing prevalence among the population. Our study led us to determine the demographic predictors of comorbid ADHD in patients with autism, which we believe can help physicians in the early diagnosis and management. Due to the nature of the disease and comorbidities, there is a critical need to invest further in the versatile and tailored care of the patients.
